# Development of image quality assurance measures of the ExacTrac localization system using commercially available image evaluation software and hardware for image‐guided radiotherapy

**DOI:** 10.1120/jacmp.v15i6.4877

**Published:** 2014-11-08

**Authors:** Dennis N. Stanley, Nikos Papanikolaou, Alonso N. Gutierrez

**Affiliations:** ^1^ Department of Radiation Oncology The University of Texas Health Science Center San Antonio San Antonio TX USA

**Keywords:** ExacTrac, IGRT, quality assurance

## Abstract

Quality assurance (QA) of the image quality for image‐guided localization systems is crucial to ensure accurate visualization and localization of target volumes. In this study, a methodology was developed to assess and evaluate the constancy of the high‐contrast spatial resolution, dose, energy, contrast, and geometrical accuracy of the BrainLAB ExacTrac system. An in‐house fixation device was constructed to hold the QCkV‐1 phantom firmly and reproducibly against the face of the flat panel detectors. Two image sets per detector were acquired using ExacTrac preset console settings over a period of three months. The image sets were analyzed in PIPSpro and the following metrics were recorded: high‐contrast spatial resolution ($f_{30},f_{40},f_{50}$ (lp/mm)), noise, and contrast‐to‐noise ratio. Geometrical image accuracy was evaluated by assessing the length between to predetermined points of the QCkV‐1 phantom. Dose and kVp were recorded using the Unfors RaySafe Xi R/F Detector. The kVp and dose were evaluated for the following: Cranial Standard (CS) (80 kV,80 mA,80 ms), Thorax Standard (TS) (120 kV,160 mA,160 ms), Abdomen Standard (AS) (120 kV,160 mA,130 ms), and Pelvis Standard (PS) (120 kV,160 mA,160 ms). With regard to high‐contrast spatial resolution, the mean values of the $f_{30}$ (lp/mm), $f_{40}$ (lp/mm) and $f_{50}$ (lp/mm) for the left detector were 1.39±0.04,1.24±0.05, and 1.09±0.04, respectively, while for the right detector they were 1.38±0.04,1.22±0.05, and 1.09±0.05, respectively. Mean CNRs for the left and right detectors were 148±3 and 143±4, respectively. For geometrical accuracy, both detectors had a measured image length of the QCkV‐1 of 57.9±0.5mm. The left detector showed dose measurements of 20.4±0.2μGy(CS), 191.8±0.7μGy(TS), 154.2±0.7μGy(AS), and 192.2±0.6μGy(PS), while the right detector showed 20.3±0.3μGy(CS), 189.7±0.8μGy(TS), 151.0±0.7μGy(AS), and 189.7±0.8μGy(PS), respectively. For X‐ray energy, the left detector (right X‐ray tube) had mean kVp readings of 81.6±0.5(CS), 122.5±0.5(TS), 122.0±0.8(AS), and 122.1±0.7(PS), and the right detector (left X‐ray tube) had 81.6±0.5(CS), 120.8±0.5(TS), 120.9±0.6(AS), and 121.3±0.7(PS). Run charts were created so that each parameter could be tracked over time and the constancy of the system could be monitored. A methodology was developed to assess the basic image quality parameters recommended by TG‐142 for the ExacTrac system. The ExacTrac system shows a consistent dose, kVp, high‐contrast spatial resolution, CNR, and geometrical accuracy for each detector over the evaluated timeframe.

PACS number: 87.10.‐e

## I. INTRODUCTION

Image‐guided radiation therapy (IGRT) requires a high standard of image quality assurance (QA) in order to ensure better localization and identification of target volumes. In principle, IGRT offers many advantages to nonimage‐guided radiation delivery in that there is enhanced delivery accuracy of precise volumetric dose distributions through the use of volumetric or planar X‐ray imaging localization techniques. The use of these modalities can enable the visualization and identification of the target volume directly or via a surrogate on both an inter‐ and intra‐fraction basis.^1^, ^2^ Clinically, IGRT allows for a reduced patient‐specific PTV margins due to the monitoring of the target volume throughout treatment,^1^, ^3^, ^4^ as well as allowing for the assessment of anatomical changes over a course of treatment.^(5)^ Additionally, the success of adaptive radiotherapy techniques intrinsically requires robust and consistent image quality for segmentation and dose calculation.^6^, ^7^

With the clinical validation of IGRT and the commercial availability of IGRT systems, image‐guided radiotherapy equipment has been rapidly integrated into clinics. To ensure correct functionality, a robust QA program should be implemented such that image quality is maximized and radiation imaging dose is minimized. An American Association of Physicists in Medicine (AAPM) Task Group has discussed and addressed the capabilities of many of these imaging systems in full detail.^(8)^ More specifically, AAPM Task Group 179 assessed the basic principles of image quality for IGRT utilizing CT‐based technologies.^(6)^ Within the report, the imaging characteristics crucial for high quality imaging were identified. As a result, the Task Group proposed a schema in which quality metric testing, frequency, and tolerance values were recommended for CT‐based technologies. In AAPM Task Group 142, the imaging quality control schema was generalized to include electronic portal imaging devices (EPID) and radiographic kV imaging modalities.^(9)^ For the majority of the quality metrics evaluated, the suggested tolerance recommendations were defined as “baseline” and assumed to be institution‐specific.

To date, there have not been any specific QA recommendations for the imaging components of the BrainLAB ExacTrac X‐ray 6D stereotactic localization system (BrainLAB AG, Feldkirchen, Germany). Previous studies have thoroughly addressed the geometric robustness and end‐to‐end spatial accuracy of the ExacTrac system in both phantom geometries and with clinical patients.^10^, ^12^, ^13^ With this in mind, the aim of this study was to develop a methodology to quality assure solely the imaging components of an ExacTrac system using the guidelines of AAPM TG 142, assess the temporal stability of the imaging quality metrics, and provide tolerance values for the imaging quality metrics based on our institutional results.

## II. MATERIALS AND METHODS

### A.1 BrainLAB ExacTrac X‐ray stereotactic localization

**Figure 1:**
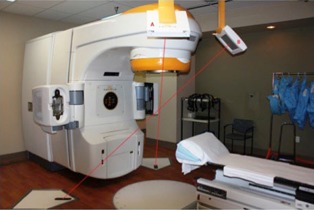
The ExacTrac X‐ray 6D image‐guided radiotherapy system of the Novalis Tx radiation delivery system is shown. The ExacTrac system is comprised of two X‐ray tubes and two flat panel detectors in the crossfire geometry.

The ExacTrac X‐ray 6D image‐guided radiotherapy system is an integration of three different subsystems: an infrared positioning system, a dual kV planar imaging system, and a 6D robotic couch. Figure 1 shows the system evaluated in this study with the dual kV planar imaging system labeled in red with an A and B. The ExacTrac system comes equipped with two ceiling mounted 25.5cm×25.5cm aSi flat panel detectors^8^, ^14^ and two corresponding kV X‐ray tubes that project medially, anteriorly, and inferiorly^(15)^ at oblique angles (45° from the mid‐sagittal plane of the accelerator^8^, ^14^) from the standard treatment position. The ExacTrac system is different with respect to traditional X‐ray systems in that: a) it has a large source‐to‐isocenter and source‐to‐detector distance (2.34 and 3.62 m, respectively^(8)^); b) the detectors and X‐ray tubes are both in a fixed position; and c) the X‐rays project in an oblique direction relative to the patient.^(16)^ Each acquired image has pixel dimensions of 512×512.^(8)^ The ExacTrac system has a geometrical accuracy of 1.5 mm in a phantom, as demonstrated by Yan et al.^(17)^ The ExacTrac Control panel has 10 different preset imaging settings, but also allows for manual changes to mA, kV, ms, and detector field size to achieve desired image quality.^(8)^ After images are acquired, they are then locally stored on the ExacTrac treatment console and can be manually exported via DICOM.

### A.2 QCkV‐1 phantom and PIPSpro V4.5.0.2

**Figure 2:**
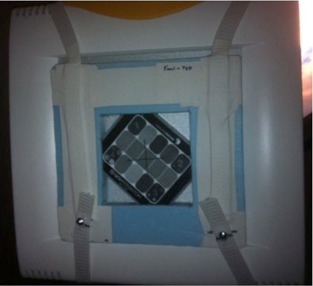
The QCkV‐1 phantom (note the various regions within the phantom used to assess image quality parameters) is shown affixed onto the face of one of the two flat panel detectors using an in‐house jig. The jig was developed to ensure accurate centering and repositioning of the QCkV‐1 phantom by indexing to the bottom corner of the flat panel detector.

The PIPSpro Quality Assurance package (Standard Imaging, Middleton, WI) was used in this study to analyze the specific image quality parameters. PIPSpro was chosen because it has a dedicated kV X‐ray phantom (QCkV‐1 phantom) and software tracking capabilities, and for its widespread use for TG‐142 EPID imaging. Using PIPSpro and the QCkV‐1 phantom, the following TG‐142 imaging metrics can be measured and analyzed: high contrast spatial resolution, contrast‐to‐noise ratio, total noise, and geometric scaling. Figure 2 shows the QCkV‐1 phantom affixed onto the face of the flat panel detector using an in‐house jig. The jig was developed to ensure accurate centering and repositioning of the QCkV‐1 phantom by indexing to the bottom corner of the flat panel detector. The QCkV‐1 phantom has 11 different regions of interest that contain line pair patterns and materials of varying densities.^(3)^ Having these different regions of the QCkV‐1 phantom allows the PIPSpro software to evaluate, store, and track the image quality parameters over time. The current version (Version 5.0) of PIPSpro software allows the user to either: 1) acquire a flood field and an image of the QCkV‐1 phantom, or 2) acquire two sequential images of the QCkV‐1 phantom for analysis. In this study, the images were evaluated using the two sequential images method, as our clinical PIPSpro software was Version 4.5.

### A.3 Unfors RaySafe Xi R/F detector

**Figure 3:**
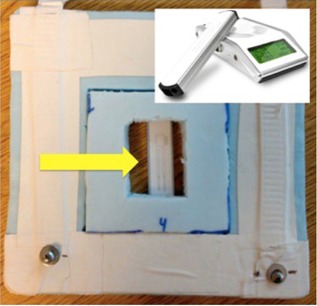
The Unfors RaySafe Xi R/F detector (denoted by yellow arrow) is shown affixed into the in‐house jig along with a customized insert. The jig and insert were developed to ensure accurate centering and repositioning of the R/F detector by indexing to the bottom corner of the flat panel detector. The top right corner of the image shows the Unfors RaySafe Xi base and R/F detector.

The Unfors RaySafe Xi (Unfors RaySafe AB, Billdal, Sweden) is a comprehensive system that offers a variety of detectors to perform multiparameter measurements on all X‐ray modalities. The system is composed of a base unit and multiple detectors that are ADCL‐certified. In this study, the R/F detector was used in conjunction with the base unit — as shown in Fig. 3. The R/F detector is a small, lightweight, and portable detector capable of measuring kVp, dose, dose rate, pulse, pulse rate, dose/frame, time, HVL, total filtration, and waveforms simultaneously. For the purposes of this study, the parameters evaluated were the dose and kVp.

A jig was constructed to hold the QCkV‐1 phantom firmly and reproducibly against the face of the ExacTrac aSi flat panel detectors. The device was designed so that it offered minimal interference with the X‐rays as well as minimal stress on the mounted detector. Figure 2 shows the QCkV‐1 phantom affixed to the detector face by this device. Two image sets (four total images) were acquired in a phantom QA patient using the ExacTrac preset console setting, Cranial Standard (CS). The two image sets were analyzed in PIPSpro and the following metrics were recorded: high‐contrast spatial resolution ($f_{30},f_{40},f_{50}$), image noise, contrast‐to‐noise ratio (CNR), and geometric image accuracy.

### B. High contrast spatial resolution, contrast to noise ratio (CNR) and total noise

PIPSpro uses the regions of different line pairs of the QCkV‐1 phantom in order to determine the high‐contrast spatial resolution of the acquired image. Each set of images has three separate values of the high‐contrast spatial resolution ($f_{30},f_{40},f_{50}$ (lp/mm)) which represent the frequencies at 30%, 40%, and 50% maximum of the relative modulation transfer function (RMTF). This method was originally purposed by Droege^(18)^ and the specific equations used were further expanded by Rajapakshe and Luchka.^(19)^ Each region of line pairs for each image is evaluated and then averaged with the other image in the image set to determine the value for the high‐contrast spatial resolution. While other QA packages may require the user to directly count and record the number of line pairs that are visible, the use of the $f_{30},f_{40}$, and $f_{50}$ parameters in regard to the RMTF offers another potential evaluation metric to increase measurement consistency by eliminating user subjectivity in the evaluation of hard‐contrast resolution. Both line pair counting and RMTF have a clinical relevance for the evaluation of the high‐contrast spatial resolution, but for the sake of consistency, reproducibility, and commercial availability, the RMTF method was chosen in this study.

**Figure 4:**
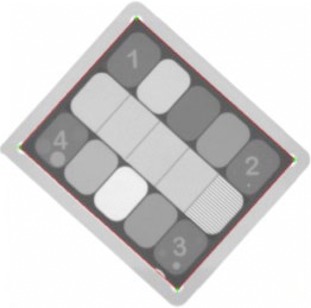
A sample ExacTrac image of the QCkV‐1 phantom. The inner boundary of the phantom is defined by the four points that dictate the correct regions of interest to evaluate. The geometric accuracy of the image was evaluated by measuring the distance between corner 1 and 2.

The process for evaluating CNR is similar to that of high‐contrast spatial resolution except PIPSpro evaluates the densities of the different materials in each region in an individual image. After comparing the images individually, the same regions of each image are compared against each other in order to determine the noise difference. Using these reference measurements, PIPSpro then compares the recorded CNR and noise for both images and records an average. The total noise for the combined images is calculated by comparing the two evaluated images. After the set of images are acquired and stored on the treatment machine, they are manually exported and saved such that PIPSpro can process the images. In the PIPSpro platform, the images are imported and the various region of interest are denoted. Figure 4 shows a sample ExacTrac image of the QCkV‐1 phantom with the inner boundary of the phantom contoured. PIPSpro will evaluate the high‐contrast spatial resolution, CNR, and total noise, and will then produce a trending report.

### C. Geometric image accuracy

Geometric image accuracy was evaluated by using the PIPSpro image measurement tool. The physical length of the QCkV‐1 phantom was measured to be 12.5 cm by 11.5 cm. For each image acquired, the length between two predefined points was measured and recorded. The physical distance between these two points was 58 mm. The distance between the points within the phantom was used as a constancy measure.

### D. Dose

Dose and kVp were evaluated using the Unfors RaySafe Xi R/F detector. An insert for the original apparatus was designed so that the Unfors RaySafe Xi R/F detector could be reproducibly held against the face of the detector (see Fig. 3). Once the Unfors RaySafe Xi R/F detector was in place, the R/F detector was exposed. After each irradiation, the kVp (kV) and dose (μGy) were manually recorded off the Unfors RaySafe Xi base unit. To establish a comprehensive evaluation of all the clinical image settings of ExacTrac, the following settings were evaluated: Cranial Standard (80 kV,80 mA,80 ms), Thorax Standard (120 kV,160 mA,160 ms), Abdomen Standard (120 kV,160 mA,130 ms), and Pelvis Standard (120 kV,160 mA,160 ms).

## III. RESULTS

**Figure 5:**
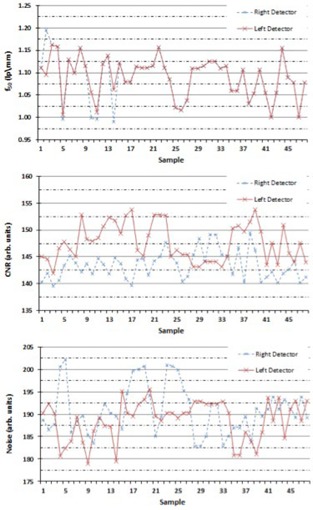
The run chart for the $f_{50}$ values (top), the CNR values (middle), and the noise values (bottom) determined by using the QCkV‐1 phantom and the PIPSpro software, for both the left and right detectors.

In all, the study evaluated 50 image sets for each detector which were acquired daily for high‐contrast spatial resolution, CNR, and noise. Thirty‐six daily measurements were acquired for dose and kVp for all the clinical settings of the ExacTrac imaging system. Table 1 shows a summary of the image quality parameters of ExacTrac using the PIPSpro software and QCkV‐1 phantom. With regard to high‐contrast spatial resolution, the mean values of the $f_{30},f_{40}$, and $f_{50}$ for the left detector were 1.39±0.04lp/mm(2.9%), 1.24±0.05lp/mm(4.0%), and 1.09±0.04lp/mm(3.7%), respectively, while for the right detector they were 1.38±0.04lp/mm(2.9%), 1.22±0.05lp/mm(4.1%), and 1.09±0.05lp/mm(4.6%), respectively. There was no statistically significant difference (p>0.01) between the left and right detectors for all high‐contrast spatial resolution parameters. Figure 5 shows the run chart of the $f_{50}$ values for both the left and right detectors.

**Table 1:** Image quality parameters of the ExacTrac localization system using PIPSpro.

	*Left Detector*	*Right Detector*
High‐Contrast Spatial Resolution (lp/mm)		
$f_{30}$	1.39±0.04	1.38±0.04
$f_{40}$	1.24±0.05	1.22±0.05
$f_{50}$	1.09±0.04	1.09±0.05
Low‐Contrast Resolution		
CNR	148±3	143±4
Noise	189±4	191±5
Geometrical Accuracy		
Length (mm)	57.9±0.5	57.9±0.5

Note: Parameter values are mean values of 50 measurements.

For the CNR and noise, the mean values for the left detector were 148±3(2.0%) and 189±4(2.1%), respectively, while the mean values for the right detector were 143±3(2.8%) and 191±5(2.6%), respectively (see Table 1). There was a statistically significant difference (p<0.01) between the left and right detectors for the CNR, but no statistically significant difference for the noise (p=NS). Figure 5 shows the run chart for the CNR and noise values, respectively, for both the left and right detectors. For geometric accuracy, both detectors had a measured image length of the QCkV‐1 of 57.9±0.5mm. There was no statistically significant difference (p>0.01) between the left and right detectors for the geometric accuracy.

Table 2 shows the dosimetric property results of the ExacTrac localization system using the Unfors RaySafe Xi R/F detector. The mean dose measurement results for the left detector were 20.4±0.2μGy(CS), 191.8±0.7μGy(TS), 154.2±0.7μGy(AS), and 192.2±0.6μGy(PS), while the mean dose measurement results for right detector were 20.3±0.3μGy(CS), 189.7±0.8μGy(TS), 151.0±0.7μGy(AS), and 189.7±0.8μGy(PS). For the dose measurements, there was no statistically significant difference between the left and right detectors for the selected anatomical site settings. For X‐ray energy, the mean kVp measurement results for the left detector were 81.6±0.5(CS), 122.5±0.5(TS), 122.0±0.8(AS), and 122.1±0.7(PS), while the mean kVp measurement results for the right detector were 81.6±0.5(CS), 120.8±0.5(TS), 120.9±0.6(AS), and 121.3±0.7(PS). Similarly to the dose measurements, there was no statistically significant difference in kVp between the left and right detectors for the selected anatomical site settings. In general, the measured kVp values for all anatomical site settings were, on average, 2.0% high relative to the kVp preset values.

**Table 2:** Dosimetric properties of the ${\text{ExacTrac}}^{®}$ localization system using the Unfors RaySafe Xi R/F.

	*Left Detector*	*Right Detector*
Dose (μGy)		
Cranial Standard	20.4±0.2	20.3±0.3
Thorax Standard	191.8±0.7	189.7±0.8
Abdomen Standard	154.2±0.7	151.0±0.7
Pelvis Standard	192.2±0.6	189.7±0.8
Energy (kVp)		
Cranial Standard	81.6±0.5	81.6±0.5
Thorax Standard	122.5±0.5	120.8±0.5
Abdomen Standard	122.0±0.8	120.9±0.6
Pelvis Standard	122.1±0.7	121.3±0.7

Note: Parameter values are mean values of 36 measurements.

**Figure 6:**
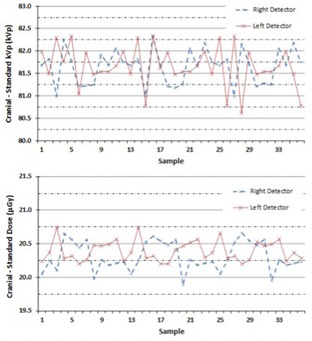
The run chart for the kVp values (top) and for the dose values (bottom) determined by using the Unfors RaySafe Xi R/F detector, for both the left and right detectors.

Figure 6 shows a run chart of both the dose and kVp measurements for the Cranial Standard setting of the ExacTrac system. The run charts were created in order to characterize the stability of the system and to help establish tolerance levels. The Cranial Standard clinical setting was selected as it was representative of the other clinical anatomical settings. In general, all clinical settings evaluated showed consistent reproducibility for both dose (mean σ=0.53%) and kVp (mean σ=0.58%) over the time period evaluated.

## IV. DISCUSSION

With the growing prevalence of IGRT treatments, the ability to determine and monitor the stability of imaging systems is important to ensure consistency of the overall imaging quality. This is of utmost significance when the imaging is used for adaptive radiotherapy, which places more restrictive constraints on the image quality over time due to its use in dose calculations. AAPM TG‐142 aids in this concern by recommending a set of annual, monthly, and daily QA requirements for different imaging systems including those that are based on planar radiography, such as the ExacTrac system. Unfortunately, the tolerance values for the various quality metrics to be examined under TG‐142 are not defined and are left to the discretion of the institution. This study was performed to develop a methodology using commercially available devices and software that allow users to establish an imaging QA program for an ExacTrac system which is TG‐142 compliant. In addition, the study serves to report our institutional tolerance values of key quality metrics of our ExacTrac system over the evaluation time period.

While there are different methods of establishing baselines and tolerance values for the various quality metrics, our institutional goal was to first create a methodology and then acquire measurements using this methodology. The baseline values were determined as the mean values of the quality metrics acquired over the course of three months. Tables 1, 2 serve to summarize the mean values and their respective standard deviation of these quality metrics. By using run chart analysis, the stability of each specific quality metric could be evaluated. Moreover, the tolerance values to determine a “warning level” and “out of tolerance level” were then defined by setting the threshold at the one sigma level and two sigma level, respectively. With this, our institution developed an imaging QA schedule that is based on the TG‐142 recommendations for our ExacTrac system. Table 3 shows our institutional imaging QA schedule for the ExacTrac system based on annual, monthly, and daily imaging QA tasks. It is important to note that the tolerance values set forth in Table 3 are strictly based on the stability analysis of our ExacTrac system using the specific methodology and measurement equipment. Although, in principle, the tolerance values for other ExacTrac systems may be similar to those in Table 3, they should be verified by a qualified medical physicist. Nevertheless, our tolerance values may be used as initial values for new QA programs and can be adjusted after the temporal response of a specific system is established. It should also be noted that the PIPSpro QA package and the Unfors dosimeter system can and have been applied to other similar planar radiographic imaging modalities like the Varian On‐Board Imager (OBI) and Elekta X‐ray Volume Imaging (XVI).^(20)^ This method is not exclusive of the ExacTrac system and can also be utilized as a way to establish institutional baselines for other kV and MV imaging modalities with the appropriate phantoms and setup. Furthermore, other commercially available software and hardware packages are available and may be substituted accordingly.

**Table 3:** Our institutional imaging QA recommendations for ExacTrac localization system.

			*Suggested Tolerance Level (Percent of Baseline)*	
*Frequency*	*Quality Metric*	*Quality Check*	*Warning Level*	*Out of Tolerance*
Daily	Safety	Warning lights	Functional	
	System operation and accuracy	phantom localization and repositioning with couch shift	±1mm	
Monthly	Geometric	Scaling and Geometrical Accuracy	±0.5mm	±1mm
	Image quality	Spatial Resolution	>4%	>8%
		Contrast to Noise Ratio	>3%	>5%
		Total Noise	>3%	>5%
Annual	Imaging quality	Imaging Dose	>1%	>2%
		Imaging Energy	>1%	>2%
	Imaging system performance	X‐ray generator performance	Functional	

The study does have a few limitations that should be mentioned. Since the current version of PIPSpro relies on the evaluation of two sequential images, an error will be introduced if the two images are not acquired at exactly the same spatial position on the face of the detector — this was a key reason for the development of the positioning jig seen in Fig. 2. Since the analysis regions of interest (ROI) in the PIPSpro software are relatively small, any discrepancy in the congruence between the two images will greatly increase the noise while simultaneously degrading the CNR and, potentially, the high‐contrast spatial resolution. Great care was taken in this study to eliminate or minimize this effect in our analysis. In the latest release of PIPSpro (v5.0.3), image analysis is done with one image and an open empty flood field. This new workflow was established to eliminate any errors introduced from the use of two sequential images. Furthermore, it should be noted that the dose and energy values were evaluated at the face of the detector and not at isocenter where a patient is most likely to be located. This was a purposeful decision since this study was interested in providing a method for establishing the dose stability of ExacTrac imaging system and not the specific dose to the patient during imaging.

## V. CONCLUSIONS

A methodology was developed to assess the basic imaging parameters for the ExacTrac localization system using commercially available imaging QA phantoms and software, as well as a diagnostic radiation dosimeter. The ExacTrac system shows a consistent and stable dose, kVp, high‐contrast spatial resolution, CNR, and geometric accuracy for both the left and right detectors over a three‐month period of daily acquisitions. No major significant differences were noted between the left and right imaging detectors of the ExacTrac system. Using the results of the study, an imaging QA schedule and suggested tolerance values for the ExacTrac system, based on the TG‐142 guidelines, was developed using daily, monthly, and annual QA tasks.
